# Double burden of malnutrition in persons with obesity

**DOI:** 10.1007/s11154-020-09578-1

**Published:** 2020-08-06

**Authors:** Rocco Barazzoni, Gianluca Gortan Cappellari

**Affiliations:** Department of Medical, Surgical and Health Sciences, University of Trieste, Trieste, Italy

**Keywords:** Obesity, Sarcopenia, Sarcopenic obesity, Malnutrition

## Abstract

A paradoxical double challenge has emerged in the last decades with respect to nutrition and nutrition-related clinical conditions. Hunger-related undernutrition continues to represent an unacceptable burden, although its prevalence has been encouragingly reduced worldwide. On the other hand, the prevalence of overweight and obesity, defined as fat excess accumulation with negative impact on individual health, has dramatically increased due to increasingly pervasive obesogenic lifestyle changes. Undernutrition and obesity may coexist in world regions, Countries and even smaller communities and households, being referred to as double burden of malnutrition. It is however important to point out that fat accumulation and obesity may also induce additional nutritional derangements in affected individuals, both directly through metabolic and body composition changes and indirectly through acute and chronic diseases with negative impact on nutritional status. In the current narrative review, associations between fat accumulation in obesity and malnutrition features as well as their known causes will be reviewed and summarized. These include risk of loss of skeletal muscle mass and function (sarcopenia) that may allow for malnutrition diagnosis also in overweight and obese individuals, thereby introducing a new clinically relevant perspective to the obesity-related double burden of malnutrition concept.

## Undernutrition and obesity: the double burden

Undernutrition due to lack of sufficient food intake, both in terms of calorie and specific micronutrient supply, has been the main survival challenge for humans throughout evolution [1, 2]. Natural disasters, wars and famine have periodically limited food availability in various parts of the world, with substantial consequences on nutritional status and health, and ultimately limited survival. Although these challenges are unfortunately far from being eliminated, their global burden has been slowly reduced in recent decades by economic, technological and social progress. On the other hand, and perhaps not surprisingly, the same profound social-economic changes have led to a substantial increase in the prevalence of obesity, as defined by increased adiposity with negative impact on patient health that is commonly diagnosed by body mass index (BMI) above 30 kg/m^2^ [3–5]. Indeed profound lifestyle changes with sedentary habits and high-calorie dietary intake causing positive energy balance may often combine with genetic predisposition, from evolutionary pressure selecting thrifty genes and phenotypes that favor energy intake and fat accumulation over energy dissipation at any given level of food availability [6, 7, 3, 4]. Combined overweight (BMI > 25 kg/m^2^) and obesity currently affect a majority of the general population in several parts of the world, with even higher prevalence in middle- and elderly age groups [8, 6, 9].

Based on the above observations, new complex nutritional challenges have emerged in recent times, with epidemiological shifts and coexistence of under- and overnutrition. The latter concept has indeed been recently described under the definition of “double burden of malnutrition”, indicating that both under- and overnutrition are commonly observed not only at global level but may indeed coexist in the same world regions and often in the same Countries and communities. Coexistence of seemingly opposite nutritional patterns may obviously partly reflect social and economic inequalities, but additional important reasons should be pointed out. In particular, enhanced life expectancy is increasing older adult population worldwide, and elderly individuals are commonly more prone to undernutrition due to multifactorial reasons that include psychological, social and health-related factors [10–12]. Even more intriguingly, the double burden of malnutrition concept is being proposed to also extend to individuals. Indeed overweight and obesity are recognized to be associated with nutritional deficiencies although awareness is currently commonly limited to impaired micronutrient levels and related clinical abnormalities, particularly under special circumstances such as pregnancy [5] and after bariatric surgery [13, 14].

## Obesity and disease-related malnutrition

The ongoing unprecedented shift in body weight paradigms is however posing larger dramatic individual, family, socio-economic and healthcare challenges [7]. Obesity is indeed a strong risk factor for metabolic diseases such as metabolic syndrome and type 2 diabetes as well as atherosclerosis and cardiovascular events, whose prevalence is accordingly rapidly increasing in the general population [15–18]. In addition, obese individuals have higher risk of developing several chronic diseases leading to end-stage organ failure as well as higher risk of cancer and infections [6, 9]. All of the above conditions may lead to acute complications and hospitalizations, and it is therefore all but surprising that obese patients are increasingly common in intensive care units (ICUs), where according to recent epidemiological data up to one-third of admissions may indeed involve obese individuals [19, 20]. Most importantly, chronic and acute diseases have a general negative impact on nutritional state, and disease-related malnutrition is recognized as a relevant cause of undernutrition in all clinical settings [21], largely through its negative impact on skeletal muscle protein-anabolic pathways and function that may be combined with variable degrees of anorexia and reduced volitional food intake [22–24]. Sarcopenia, defined as reduction of skeletal muscle mass and function, is indeed common in many disease conditions, and may be associated with excess body fat in persons with obesity, thereby introducing the concept of sarcopenic obesity [25]. Although its clinical relevance is emerging as a potential important risk factor for negative outcomes, the concept of sarcopenic obesity and, more generally, of malnutrition in obesity still needs research efforts and consensus initiatives aimed at better clarifying its definition, diagnostic criteria and treatment options [25].

## Obesity and skeletal muscle loss and dysfunction

Negative obesity- and disease-related influences on skeletal muscle mass and function maintenance are summarized below. It is increasingly clear that profound alterations in skeletal muscle metabolism may occur in obesity with potential negative impact on whole body muscle mass and function [6, 26]. Body composition consequently changes with lower lean body mass associated with high body fat, and these combined alterations are commonly reported to exert a negative impact on patient morbidity and mortality [6, 27, 28]. Various mechanisms may contribute to obesity-associated loss of skeletal muscle mass and function:

1. Intermediate and energy metabolism changes.
  - primary metabolic abnormalities and insulin resistance: although a sizable portion of the obese population is not insulin resistant, a large majority of insulin resistant persons is either overweight or obese, or it presents with excess fat accumulation in the abdominal visceral compartment [29, 30]. Substantial advances were made in the last decades in understanding the pathogenesis of insulin resistance and its profound association with excess adiposity and excess nutrient and substrate availability [31, 32]. Complex underlying mechanisms may include repeated acute exposures to glucose and fatty acid elevations at systemic and tissue levels, that may result in excess ROS production and inflammation as well as altered adipose tissue function leading to pro-inflammatory, insulin desensitizing and atherogenic endocrine signalling [33–35]. Involvement of gut nutrient-sensing hormonal systems as well as central nervous system sensors regulating appetite and intermediate metabolism has been demonstrated in the pathogenesis of obesity-associated insulin resistance [36–44]. In addition, gut microbial responses to nutrient intake and hormones have far-reaching systemic effects on tissue substrate utilization and insulin action [45, 46]. Detailed description of these mechanisms is beyond the scope of the current review. Most importantly however all the above alterations have strong catabolic potential by altering skeletal muscle protein turnover with enhanced protein breakdown and inhibition of protein synthesis [47, 48], and they may induce “anabolic resistance” in skeletal muscle, with blunted response of muscle protein synthesis to nutrients and amino acids [49–52];
  - ectopic muscle fat accumulation: muscle lipid accumulation commonly results in obesity from impaired adipose tissue expansion in the presence of excess lipid availability. Skeletal muscle fat accumulation is conversely closely associated with tissue and systemic insulin resistance [10, 48]. Mechanisms underlying metabolic lipotoxicity are complex and only partly understood, but they likely include direct pro-oxidative and inflammatory activities and accumulation of metabolically toxic moieties such as diacylglycerol and ceramides [53];
  - mitochondrial dysfunction: mitochondrial changes may not occur in obese individuals and become relatively common only in relatively late stages; their onset may however lead to excess ROS production resulting in oxidative stress and related complications that may exacerbate insulin resistance and directly favor muscle loss [48, 31, 44]. It should also be pointed out that altered mitochondrial function with impaired ATP production may directly result in impaired ability to perform contractile work with low muscle strength and endurance;
  - stem cell dysfunction: altered muscle stem cell function with excess adipocyte differentiation [54] have been described in obesity models and following fat accumulation. Muscle stem cell dysfunction may conversely directly limit skeletal muscle mass maintenance;
2. Comorbidities and treatment.
  - metabolic complications: metabolic syndrome or overt type 2 diabetes and hyperglycemia are common in insulin resistant obese individuals and their onset may exacerbate pro-oxidative and inflammatory alterations as well as mitochondrial dysfunction. These enhanced abnormalities may further promote muscle loss and functional alterations [47, 48];
  - chronic disease: chronic organ failure syndromes result in enhanced inflammation and oxidative stress through various mechanisms at tissue and systemic level, thereby also synergistically promoting muscle loss and damage [55]. Obesity directly enhances the risk for, or may be associated with organ failure syndromes including chronic heart failure, chronic kidney disease, chronic obstructive pulmonary disease and obstructive sleep apnea syndrome as well as cancer [6, 56, 9];
  - surgery: bariatric procedures are becoming increasingly common and almost invariably lead to skeletal muscle catabolism at least in the initial rapid weight loss phase characterized by profoundly negative energy balance [57, 58]; low- or very low calorie diets are associated with similar qualitative changes although to less pronounced degrees [59].
3. Lifestyle and physical inactivity: low physical activity levels play a key role in the onset of obesity and physical activity is commonly further progressively reduced with disease duration, aging and increased body weight, with direct negative impact on muscle protein turnover as well as muscle fitness [60]. Obesity-associated chronic comorbidities and organ failure syndromes may further impair muscle mass maintenance by lowering physical activity and fitness.

Based on the above observations, a multifactorial cluster of obesity-induced or obesity–associated alterations leads to skeletal muscle loss and dysfunction with low muscle strength and endurance, that are likely caused at least in part by low muscle mass, mitochondrial dysfunction with altered bioenergetics and fat accumulation with reduced muscle density. These changes become progressively more likely in patients with longer obesity duration, complications and older age, that may be per se independently associated with muscle loss and dysfunction.

## Clinical impact: Sarcopenic obesity

Skeletal muscle changes in obesity have a profound clinical impact. Obese individuals with low muscle mass and function have higher risk of developing frailty and disability, and may generally present with poorer quality of life. Importantly, low or declining muscle mass is emerging as a negative prognostic factor associated with higher morbidity and mortality in obese patients with various chronic complications and diseases and in aging [61–63, 11, 64, 65]. Obese individuals with low muscle mass or functional parameters were at higher risk of developing frailty and disability than non-obese control individuals [66, 67]. Low skeletal muscle mass or its reduction is also reported to be associated with impaired survival in obese individuals with chronic diseases [68, 69]; in obese patients with diseases ranging from gastrointestinal cancer to heart failure low muscle mass or physical capacity accordingly resulted in higher risk of dying and generally poorer outcomes [70, 68, 69]. In chronic kidney disease patients, changes in muscle mass were the major determinant of survival even independently of changes in total body weight [71].

The potential relevance of muscle mass maintenance as a priority therapeutic target in obesity is regrettably hampered by inadequate awareness. Definition and diagnosis of this condition are problematic. Definition of sarcopenic obesity may combine obesity with the geriatric concept of primary sarcopenia, a combination of low muscle mass and function (particularly strength) that almost invariably occurs in old age. While diagnostic criteria for aging-associated primary sarcopenia have been proposed [72], they are not unanimously accepted and their combination with obesity to identify obese individuals with low muscle mass and function remains problematic. A recent systematic review [73] clearly demonstrated high heterogeneity in sarcopenic obesity definition in available literature, making appropriate scientific conclusions and clinical approaches difficult. The European Society of Clinical Nutrition and Metabolism (ESPEN) and the European Association for the Study of Obesity (EASO) have produced joint documents calling for initiatives and action to address consensus gaps and provide practical guidance in this field [74, 75].

## Obesity, skeletal muscle and malnutrition screening, diagnosis and treatment

Overall, despite relevant practical limitations, recognizing skeletal muscle depletion or dysfunction in obese individuals by body composition measurement techniques, strength measurement or other functional tests should be a priority to alert clinicians to enhanced risk for negative outcome [76–78].

Routine nutritional screening to identify patients at risk for malnutrition is recommended in most chronic and acute disease conditions and in hospitalized patients. Validated and widely utilized screening tools include Nutritional Risk Screening-2002 (NRS-2002) [79]. Importantly, use of NRS 2002 may allow to identify nutritional risk also in obese individuals [80, 81]. A major recent advance is represented by the global consensus initiative defined as global leadership initiative on malnutrition (GLIM) [82, 83]. In this context, experts from major clinical nutrition Societies worldwide have proposed simple diagnostic criteria to identify malnutrition in all clinical settings, independently of its etiology and underlying or associated diseases. At least one phenotypic (out of three) and one pathogenetic criterion (out of two) are needed for malnutrition diagnosis. Although low BMI is one phenotypic criterion that may lead to malnutrition diagnosis, malnutrition can be diagnosed also in the presence of high BMI values if alternative phenotypic criteria are fulfilled, including low skeletal muscle mass and non-volitional weight loss [82, 83]. The GLIM criteria therefore provide a major conceptual advance in allowing for malnutrition diagnosis in the presence of high BMI and adiposity. Clinical approaches to identify obese individuals with skeletal muscle dysfunction are also increasingly implemented in the obesity community, where the inadequacy of BMI to identify and stratify clinical risk of complications and poor outcomes is being more and more appreciated. The Edmonton obesity staging system (EOSS) [84] is a recently-proposed assessment tool for obese individuals including a functional domain that largely implies the importance of preserved or compromised skeletal muscle mass and function. In general, the importance of measuring or assessing skeletal muscle mass and function and/or body composition is becoming more and more recognized and a better and widely accepted definition of sarcopenia and sarcopenic obesity are likely to improve awareness of this key feature in both researchers and clinicians.

Most importantly, major guidelines have started to take nutritional needs of obese patients into account with specific recommendations for nutritional support that may combine a limitation in calorie with high protein administration aimed at preventing loss of skeletal muscle mass and function [85, 86]. Prevention of skeletal muscle loss and dysfunction is also increasingly recognized as a relevant priority in lifestyle interventions aimed at achieving sustained weight loss [87], with adequate protein dietary intake and exercise activity being the cornerstones of treatment [87, 88].

## Conclusions: the double burden concept expanded

In conclusion, the ongoing obesity epidemics, along with population aging and increased chronic disease prevalence is profoundly modifying fundamental clinical nutrition and healthcare priorities worldwide. A true double burden of malnutrition exists beyond coexistence of obesity and undernutrition in Countries and communities. Indeed individuals with obesity may present with true malnutrition due to inability to preserve body composition and performance with loss of skeletal muscle loss and function that exert major negative influences on morbidity and survival. Recognition of this new double burden is a relevant priority in order to more effectively identify diagnostic and therapeutic tools.
